# Dominant Occipital Sinus: A Rare Anatomical Variant With Potentially Catastrophic Consequences if Unrecognized Preoperatively

**DOI:** 10.7759/cureus.76296

**Published:** 2024-12-24

**Authors:** Arman Khan, Richard Moon, Matias Costa, Daniel Casanova-Martinez, Mario Teo

**Affiliations:** 1 Medical School, University of Adelaide, Adelaide, AUS; 2 Department of Neurosurgery, Southmead Hospital, North Bristol NHS Trust, Bristol, GBR; 3 Department of Neurological Surgery, University of Texas Medical Branch, Galveston, USA; 4 Faculty of Medicine, University of Valparaiso, Valparaiso, CHL

**Keywords:** dominant occipital sinus, dural venous sinus, suboccipital craniotomy, surgical morbidity, transverse sinus hypoplasia

## Abstract

The occipital sinus is often thought of as a redundant vestigial structure in adults. However, in rare cases, it can form the dominant route of intracerebral venous drainage, with a risk of significant surgical morbidity if unrecognised. We present an illustrative case describing this anatomical variant and tailoring of a midline suboccipital craniotomy to allow resection of a fourth ventricular epidermoid tumour with preservation of a dominant occipital sinus, and a review of the published literature. A 48-year-old female patient was diagnosed with a large fourth ventricular tumour with marked diffusion restriction, consistent with an epidermoid tumour. Imaging demonstrated bilateral hypoplastic transverse sinuses and a widely patent occipital sinus draining the straight and superior sagittal sinuses into the marginal sinus. A midline posterior fossa craniotomy, C1 laminectomy, and paramedian durotomy with the division of the left marginal sinus allowed for gross total resection of the epidermoid tumour with preservation of the occipital and right marginal sinuses. Given the significant potential surgical morbidity resulting from injury or ligation of a dominant occipital sinus, as seen in the literature review, we highlight the importance of recognising anatomical variants of the dural venous sinuses preoperatively to modify surgical approaches and minimise potential complications.

## Introduction

The occipital sinus is one of the smallest dural venous sinuses, lying in the attached margin of the falx cerebelli along the internal occipital crest of the occipital bone. Tributaries from the torcula Herophili and medial transverse sinuses coalesce as the occipital sinus, and then drain into the marginal sinus at the foramen magnum and sometimes also the distal sigmoid sinuses [1]. The occipital sinus reduces in calibre during the latter half of gestation, followed usually by subsequent age-related regression [2].

The predominant route of venous outflow from the adult brain is typically via the transverse and sigmoid sinuses. If encountered during surgical exposure of the posterior fossa, the occipital sinus is commonly ligated to expose the suboccipital cerebellar surface and obex. However, it can be of critical surgical importance when variations in the dural venous anatomy are present, particularly a dominant occipital sinus. If unrecognised preoperatively, injury to dominant variant venous drainage pathways can have potentially devastating complications. Here, we describe modifications to a suboccipital craniotomy to preserve aberrant dural venous sinus drainage and allow resection of a fourth ventricular epidermoid tumour in an adult reliant on the occipital sinus for venous outflow, followed by a review of the literature.

## Case presentation

**Figure 1:**
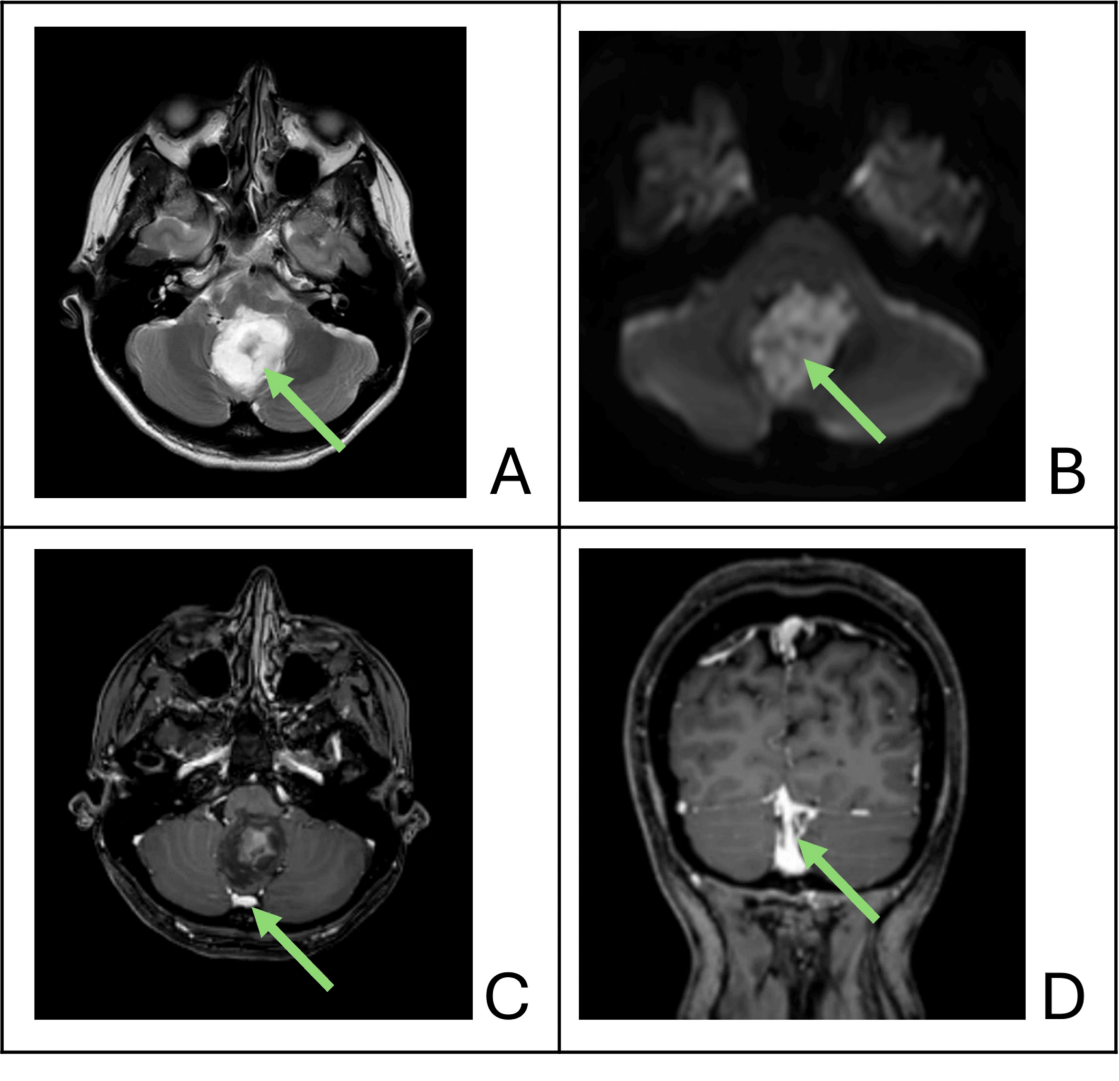
Magnetic resonance imaging of fourth ventricular tumour and dural venous sinuses (A) Axial T2 image showing hyperintense fourth ventricular lesion (arrow) with compression of the brainstem and cerebellar peduncle; (B) Axial diffusion-weighted image showing restricted diffusion (arrow) consistent with epidermoid tumour; (C) Axial T1 post-gadolinium image showing large midline occipital sinus (arrow) and non-enhancing epidermoid tumour; (D) Coronal T1 post-gadolinium image showing dominant occipital sinus (arrow) with bilateral hypoplastic transverse sinuses

A 48-year-old woman presented with a four-year history of headache, dizziness, and neck pain. Past medical history was irrelevant. Clinical examination was normal, with no impairment of motor, sensory, cranial nerve or cerebellar function. Magnetic resonance imaging (MRI) demonstrated a heterogenous, predominately cystic, T2 hyperintense 4 cm mass within the fourth ventricle. There was no enhancement but marked restricted diffusion, consistent with an epidermoid tumour (Figure 1). Bilateral hypoplastic transverse sinuses were evident, with a widely patent occipital sinus draining the straight and superior sagittal sinuses, bifurcating to join the marginal sinus at the foramen magnum and draining into the internal jugular veins bilaterally (Figure 1). There was no evidence of venous hypertension, nor enlargement of alternative venous drainage pathways.

**Figure 2:**
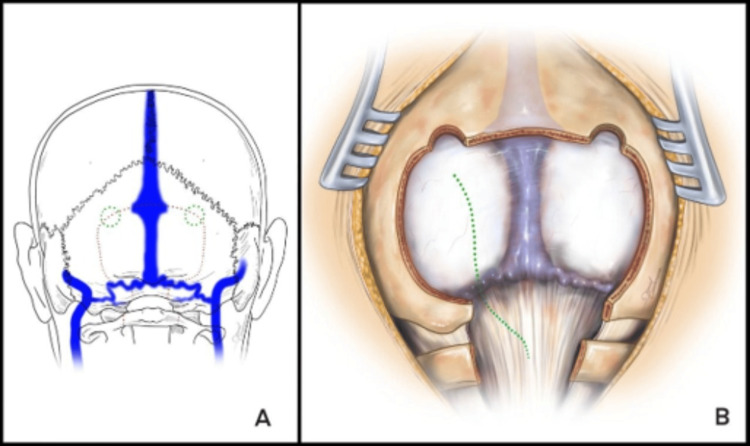
Intraoperative anatomy (A) Schematic illustration of venous anatomy and craniotomy margins showing a dominant occipital sinus draining into the marginal sinus at the level of the foramen magnum, with bilateral hypoplastic transverse sinuses at the superior craniotomy margin; (B) Illustration of visualised operative anatomy, including location of occipital and marginal sinuses as identified by Doppler ultrasound and the site of paramedian durotomy (dotted line) with division of left marginal sinus and preservation of dominant occipital and right marginal sinuses Image Credit: Dr. Daniel Casanova-Martinez

Following detailed pre-operative counselling, the patient underwent surgical resection of the epidermoid tumour with intraoperative neurophysiological monitoring of the lower cranial nerves (trigeminal to hypoglossal). A midline suboccipital craniotomy and C1 laminectomy were performed through a midline suboccipital incision. Intraoperative handheld Doppler ultrasound demarcated absent bilateral transverse sinuses and a prominent inferior occipital sinus (Figure 2). A paramedian durotomy was fashioned from the midline at the caudal extent of the exposure to the left cerebellar hemisphere, dividing the left marginal sinus and preserving the occipital and right marginal sinuses (Figure 2).

**Figure 3:**
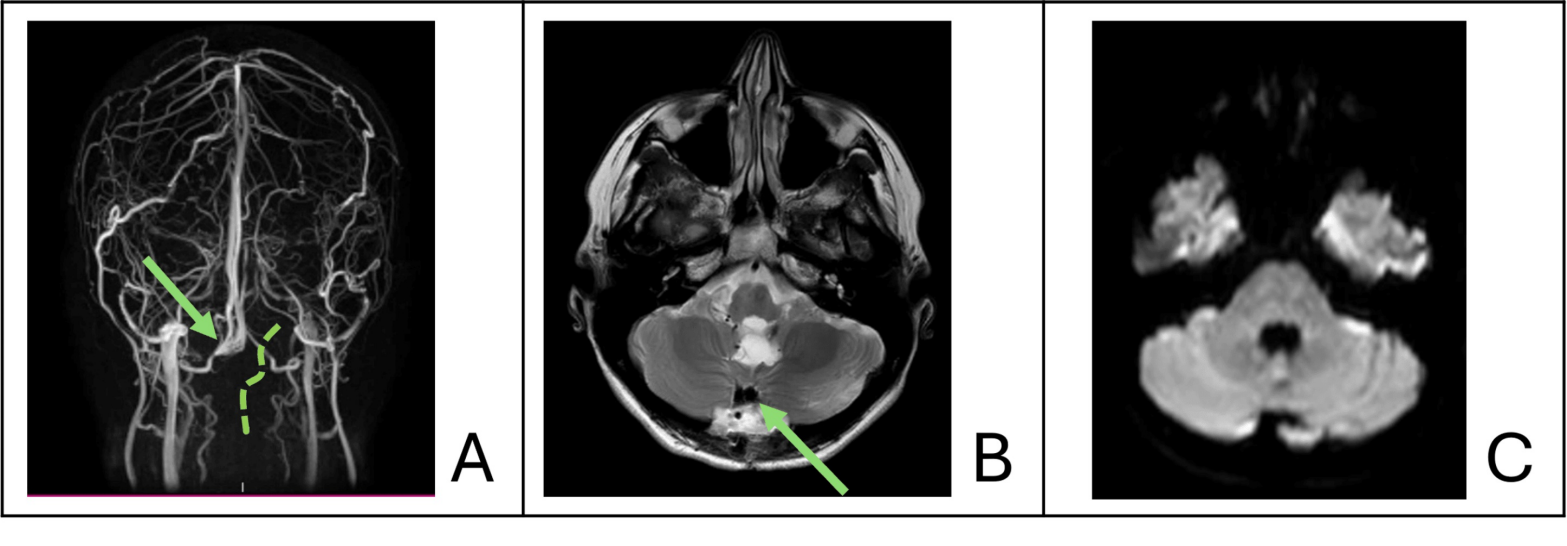
Postoperative magnetic resonance imaging (A) Coronal MR venography showing patent dominant occipital and right marginal sinuses (arrow) and ligation of left marginal sinus at site of paramedian durotomy (dotted line); (B) Axial T2 MRI showing gross total resection with patent occipital sinus flow void (arrow); (C) Axial diffusion-weighted image showing no evidence of residual epidermoid tumour

Reflection of this dural incision allowed excellent exposure of the suboccipital cerebellar surface and cisterna magna. The floor of the fourth ventricle was identified and preserved during microsurgical dissection, with stimulation mapping of the trigeminal, abducens, facial, and vagal nuclei. Gross total resection of the epidermoid tumour was achieved without complication. No postoperative neurological deficits were noted, and postoperative imaging demonstrated patent occipital and right marginal sinuses and no evidence of residual epidermoid tumour (Figure 3). Surveillance imaging five years post resection demonstrated no epidermoid tumour recurrence.

## Discussion

Literature review

A literature search was performed in Pubmed using the search term “dominant occipital sinus”, limited to English language publications. The titles and abstracts of all results were screened by the first and second authors to identify any publications reporting a suboccipital craniotomy/craniectomy encountering a dominant occipital sinus. Reports in which the venous anatomy and its relationship to surgical decision-making were not described were excluded. The reference lists of accepted reports were examined for additional articles not identified in the initial search. Given that the majority of included articles contained single case reports or limited case series, a formal quality assessment was not performed.

**Figure 4:**
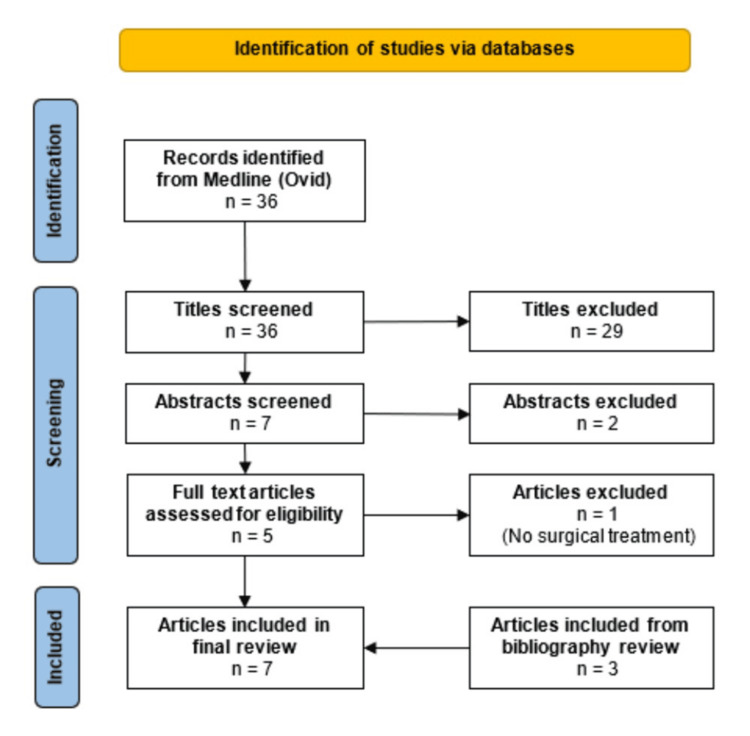
Flow chart of article selection for literature review

The literature search yielded an initial result of 36 articles. Of these, seven articles were selected for abstract screening, five for full-text screening, and four articles meeting the eligibility criteria were included in the final analysis. An additional three articles were included following the review of reference lists (Figure 4). A total of 12 cases met the inclusion criteria from the seven included articles (Table 1), including 10 female patients, with a median age of 15 years (range 3-41 years). Nine patients underwent suboccipital craniotomy due to Chiari 1 malformation, with the remaining three patients undergoing resection of posterior fossa tumours.

**Table 1:** Summary of surgical cases encountering a dominant occipital sinus from literature review OS: occipital sinus; TS: transverse sinus; SS: sigmoid sinus; MS: marginal sinus; GCS: glasgow coma scale; F: female; M: male

Author (publication year)	Age (years)/ Gender	Pathology	Venous anatomy	Surgical approach	Complications	Outcome
Hayashi et al. (2018) [3]	27F	Chiari 1 malformation	Large oblique OS, hypoplastic right TS and SS	Suboccipital craniectomy, bilateral durotomies either side of OS	Nil	Resolution of pre-operative symptoms
Kizmazoglu et al. (2017) [4]	32F	Fourth ventricular low-grade glioma	Midline OS, bilateral hypoplastic TS and SS	Suboccipital craniotomy, tailored limited durotomy	Suboptimal exposure and subtotal resection. Haemorrhage into residual tumour	Low GCS and significant cerebellar dysfunction
Tochigi et al. (2023) [5]	29F	Chiari 1 malformation with syringomyelia	Unilateral oblique OS, hypoplastic contralateral and patent ipsilateral TS	Suboccipital craniectomy, unilateral curvilinear durotomy	Nil	Reduction in syrinx
	41F	Chiari 1 malformation with syringomyelia	Unilateral oblique OS, hypoplastic contralateral and patent ipsilateral TS	Suboccipital craniectomy, unilateral curvilinear durotomy	Nil	Reduction in syrinx
	15F	Chiari 1 malformation with syringomyelia	Unilateral oblique OS, hypoplastic ipsilateral and patent contralateral TS	Fourth ventricle-subarachnoid shunt and syringo-cisternal shunt, limited dural opening from C1 to cervicomedullary junction	Nil	Reduction in syrinx
	7M	Chiari 1 malformation with syringomyelia	Bilateral oblique OS, bilateral patent TS	Fourth ventricle-subarachnoid shunt, limited dural opening from C1 to cervicomedullary junction	Nil	Reduction in syrinx
	4F	Chiari 1 malformation with syringomyelia	Bilateral oblique OS, bilateral hypoplastic TS	Fourth ventricle-subarachnoid shunt, limited dural opening from C1 to cervicomedullary junction	Massive intra-operative haemorrhage from OS injury with significant cerebellar swelling	No neurological sequelae, reduction in syrinx
	13M	Chiari 1 malformation with syringomyelia	Bilateral oblique OS, bilateral hypoplastic TS	Fourth ventricle-subarachnoid shunt, limited dural opening from C1 to cervicomedullary junction	Nil	Re-expansion of syrinx
Tyagi et al. (2021) [6]	3F	Vermian medulloblastoma	Unilateral oblique OS, Hypoplastic but patent ipsilateral TS	Midline suboccipital craniotomy and Y-shaped durotomy with ligation of OS	Cerebellar swelling and significant intraoperative haemorrhage. Partial tumour debulking, decompressive craniectomy and delayed tumour resection	Improvement in pre-operative cerebellar neurological signs
Fukai et al. (2002) [7]	10F	Vermian medulloblastoma	Midline OS, unilateral hypoplastic TS	Suboccipital craniectomy, ligation of OS	Cerebellar swelling and intra-tumoural haemorrhage	Full recovery of pre-operative neurological deficits
Champagne et al. (2018) [8]	15F	Chiari 1 malformation with syringomyelia	Midline OS, bilateral hypoplastic TS	Suboccipital craniectomy, ligation of OS and MS	Post-operative intracranial hypertension with papilloedema and VIth nerve palsy	Resolution of intracranial hypertension with acetazolamide
Omoto et al. (2020) [9]	40F	Chiari 1 malformation with syringomyelia associated with cleidocranial dysplasia	Unilateral oblique OS, bilateral hypoplastic TS, bilateral hypoplastic SS	Suboccipital craniectomy, unilateral curvilinear durotomy	Nil	Resolution of pre-operative symptoms and reduction in syrinx

Of the 12 reported cases of a dominant occipital sinus encountered during suboccipital craniotomy, nine had a unilateral occipital sinus (three midline [4,7,8], six oblique draining directly into the sigmoid sinus [3,5,6,9]) and three had bilateral oblique occipital sinuses [5] (Table 1). Eleven patients had hypoplasia of the transverse sinuses, unilaterally in six [3,5-7] and bilaterally in five [4,5,8,9]. Only six cases reported whether any hypoplasia of the sigmoid sinuses was identified, of which three had bilaterally patent sigmoid sinuses [6-8], one a unilateral hypoplastic sigmoid sinus [3], and two bilateral hypoplastic sigmoid sinuses [4,9]. A custom durotomy adapted to the venous anatomy was performed in five patients [3,5,9], a limited midline durotomy in four [4,5], and a classic Y-shaped durotomy with ligation of the occipital sinus in three [6-8].

Intra- or postoperative complications were reported in five patients (42%), including in all three cases where ligation of a dominant occipital sinus occurred [4-8]. Of these, two experienced significant cerebellar swelling and intraoperative haemorrhage (necessitating delayed tumour resection in one case), and another postoperative intracranial hypertension secondary to diminished venous outflow, requiring acetazolamide therapy [6-8]. Complications also occurred in two patients where the occipital sinus was preserved [4,5]. Poor surgical exposure and subsequent subtotal tumour excision in one patient led to haemorrhage into residual tumour and significant neurological injury, whilst injury to the occipital sinus in another case led to significant intra-operative haemorrhage and cerebellar swelling.

Discussion

In rare cases, the occipital sinus can form the dominant route of intracerebral venous drainage but is routinely ligated during a suboccipital craniotomy. We described the use of a paramedian dural incision during exposure of the cisterna magna and suboccipital cerebellar surface to preserve a dominant occipital sinus and allow gross total resection of a fourth ventricular epidermoid tumour. The literature review highlighted significant morbidity following ligation or injury to a dominant occipital sinus and a range of different approaches to ameliorate this risk.

The occipital sinus is derived from the medial channel of the embryonic posterior dural plexus and gradually reduces in calibre during the second half of gestation so that only one midline venous channel is present within the falx cerebelli at term [10]. Regression is then assumed to continue up until two years of age, evidenced by the differing incidence seen in paediatric and adult populations. The occipital sinus has been reported in up to 100% of patients in cadaveric neonatal studies, then reduces in frequency in infant studies due to increased venous drainage into the vertebral venous plexus, through to an incidence of 9-57% in adult radiological series [5,11-16]. It has been described more frequently in cadaveric studies than radiological, theorised to be due to technical limitations of imaging flow in small calibre sinuses [17]. The relationship between the transverse sinuses and a persistent occipital sinus is key, as this determines whether a persistent occipital sinus may form the dominant route of venous drainage. Magnetic resonance (MR) venography has demonstrated unilateral transverse hypoplasia in up to 21% of adults (left more common than right), and aplasia in 4.1% [15,18]. Bilateral hypoplastic transverse sinuses are comparatively rarer, reported in 1.6% of cases [18]. It is interesting that some studies have determined a dominant occipital sinus on the basis of size relative to the sigmoid sinus alone, without reference to transverse sinus patency [5].

The occipital sinus connects the medial portion of the transverse sinuses and torcula Herophili to the marginal sinus or distal sigmoid sinuses. However, many different anatomical variations have been described, including a single midline sinus draining to the marginal sinus, multiple sinuses draining from the torcula, or an oblique sinus draining uni- or bilaterally into the sigmoid sinus directly [16,19]. The different patterns of an oblique occipital sinus have been further stratified according to the risk of venous congestion and major haemorrhage, with the highest risk ascribed to those patients with bilateral oblique occipital sinuses in the presence of bilateral hypoplastic/aplastic transverse sinuses [5,19]. The venous anatomy and associated peri-operative risk are obviously critical to surgical planning, for example, in the context of symptomatic Chiari 1 malformation, the highest risk configuration of a dominant occipital sinus led the authors to recommend avoidance of duroplasty and insertion of a fourth ventricular-subarachnoid shunt instead [5].

Multiple different methods to open the dura and expose the suboccipital cerebellar surface and cisterna magna are described within the studies identified in our literature review. These included bilateral durotomies on either side of the occipital sinus, a unilateral curvilinear durotomy, and a more limited dural opening below the cervicomedullary junction [3,5,20]. The nature of the dural incisions often entails a more limited surgical exposure, although we demonstrate the ability to safely achieve gross total resection of a midline lesion through a unilateral approach. Whilst maximising the possible surgical exposure is important to allow safe microsurgical dissection, this should not be at the expense of putting undue tension on the occipital sinus and risking sinus injury, since this can equally be associated with massive intra-operative haemorrhage and cerebellar swelling [5]. There is clearly no one-size-fits-all surgical approach for these challenging cases, and we do not advocate any one particular technique, but tailoring of the durotomy following careful study of individual anatomy.

It is difficult to gauge the true incidence of a dominant occipital sinus due to its rarity, the differences between radiological and cadaveric studies, and the variation in the definition of when an occipital sinus is dominant in different studies. It is therefore hard to accurately stratify the relative risk of haemorrhage or venous congestion with different anatomical variants. Angiographic studies, such as CT or MR venography, do not provide a quantitative assessment of flow, limiting our ability to fully delineate the venous drainage pattern or predict the impact of complete or relative obstruction. It is likely that both persistent and dominant occipital sinuses are encountered more commonly during posterior fossa surgery than have been identified in the literature review, and so we highlight the anatomical variations and associated risks to further inform surgical planning.

## Conclusions

Dominant venous drainage via the occipital sinus is a rare pattern. Injury or ligation of a dominant occipital sinus is associated with significant intraoperative haemorrhage and cerebellar swelling. Given the risk of severe morbidity during posterior fossa surgery, if a dominant occipital sinus is unrecognised, we reiterate the need for a thorough preoperative evaluation of venous anatomy. Carefully tailored surgical approaches can allow the preservation of aberrant venous drainage with sufficient intraoperative exposure for safe resection of fourth ventricular lesions.
